# Torsion of Uterus Didelphys at Term: Once-in-a-Lifetime Experience for an Obstetrician

**DOI:** 10.7759/cureus.31996

**Published:** 2022-11-28

**Authors:** Mousumi D Ghosh, Vinita Singh, Mamta R Datta

**Affiliations:** 1 Obstetrics and Gynecology, Tata Main Hospital, Jamshedpur, IND

**Keywords:** obstetrician, transverse lie, uterus didelphys, torsion, pregnancy

## Abstract

Torsion of the gravid uterus is very rare in obstetric practice. We report a case of torsion in the uterus didelphys at term which is rare and a lifetime experience for an obstetrician.

The patient, a 25-year-old gravida 2 para 1 was admitted to the labor ward at 37 weeks and six days of gestation with abdominal pain. Her previous delivery was a caesarean section performed four years back. She was taken to the operating room for an emergency caesarean section for fetal distress and the lie was transverse. On entering the peritoneal cavity, we found an engorged infundibulopelvic ligament with the fallopian tube and ovary covering the lower segment of the uterus. The baby was successfully delivered by breech extraction. Due to uterine torsion of more than 180 degrees, the posterior surface of the uterus was placed anteriorly, and the incision was made on the posterior surface of the uterus. There was a hemi uterus on the left side of the pelvic cavity with the fallopian tube and ovary attached to it; a diagnosis of uterus didelphys was made. The diagnosis of uterine torsion is intraoperative and prompt and timely decision by surgeons is crucial. We had favorable maternal and fetal outcomes in this rare and interesting case. The diagnosis, though rare, should be kept in mind in all cases of abdominal pain during pregnancy, especially in those with malpresentation.

## Introduction

Torsion of the uterus is a rare condition which is infrequently encountered in clinical practice. Encountering a patient with an unusual and rare combination of torsion in the uterus didelphys at term makes it a lifetime experience for an obstetrician [1]. There are very few published cases in the literature till date.

## Case presentation

A 25-year-old, gravida 2 para 1 was admitted to the labour ward with abdominal pain at 37 weeks and six days of gestation. Her last childbirth was by caesarean section four years back. Her antenatal period was uneventful with regular check-ups as per schedule. She had transverse lie and was planned for elective repeat caesarean section at 39 weeks.

**Figure 1:**
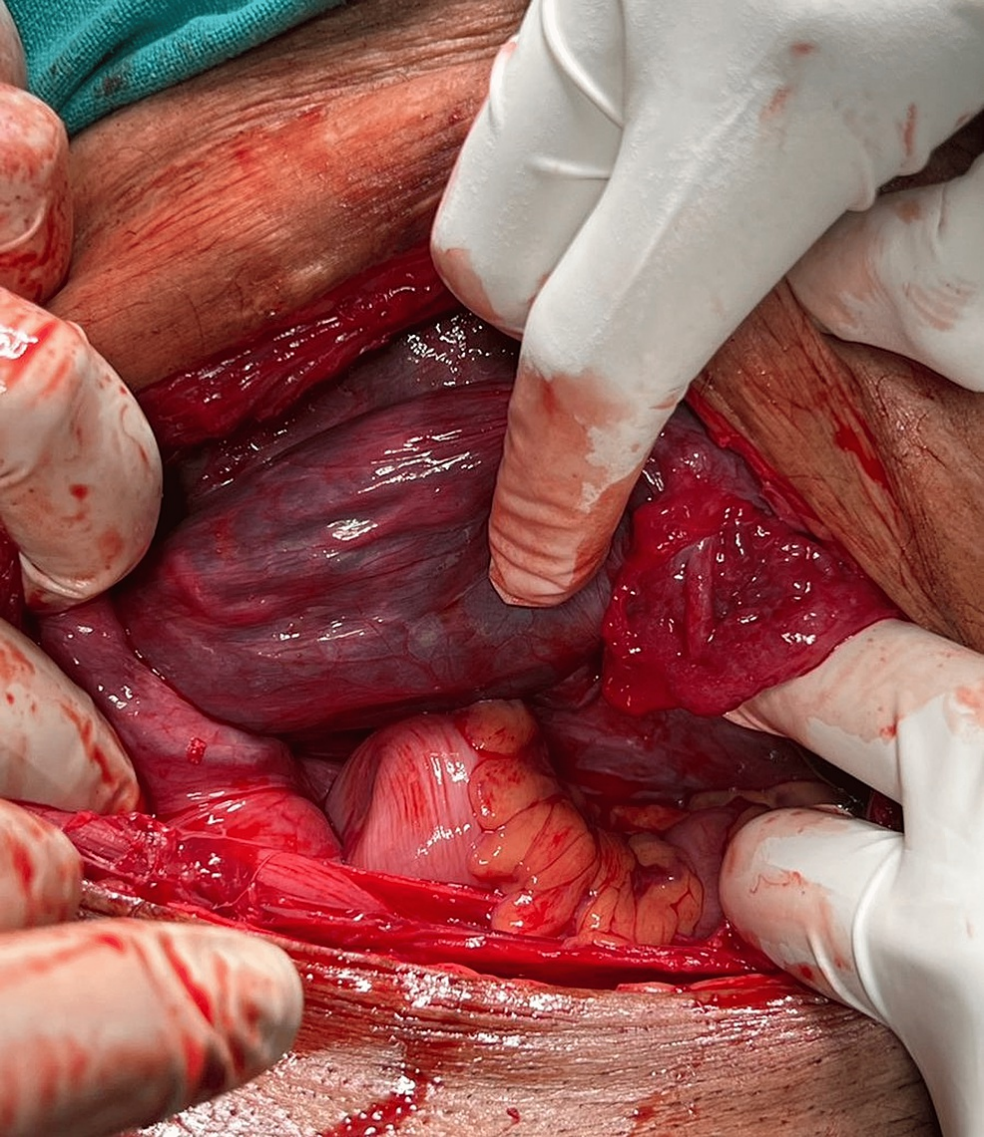
Stretched infundibulopelvic ligament with the fallopian tube occupying the lower segment of the uterus

On examination, there were mild uterine contractions and the lie was transverse. Ultrasound was done which showed a single live fetus with an estimated fetal weight of 3.2 kg. The placenta was anterior and low-lying, ending 1 cm above the internal os. A mass of 12 x 7.6 cm was found in the pelvis which was suspected to be a cervical myoma. Cardiotocography indicated fetal distress and she was prepared for an emergency caesarean section. The abdomen was opened by Pfannenstiel incision. On entering the peritoneal cavity, we were surprised to see the engorged infundibulopelvic ligament with the fallopian tube and ovary stretched across the operative field (Figure 1). 

**Figure 2:**
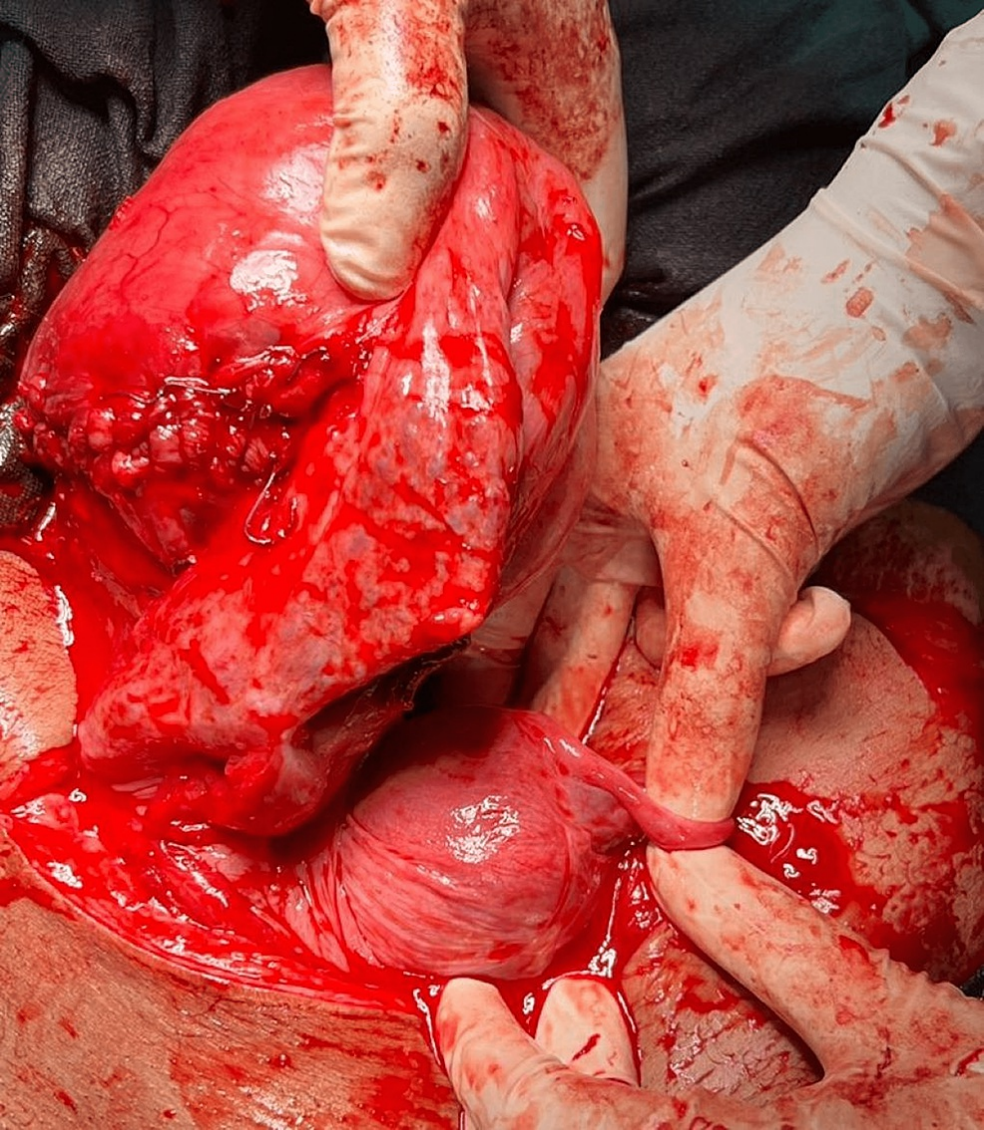
Uterus didelphys with incision over posterior surface of the uterus

**Figure 3:**
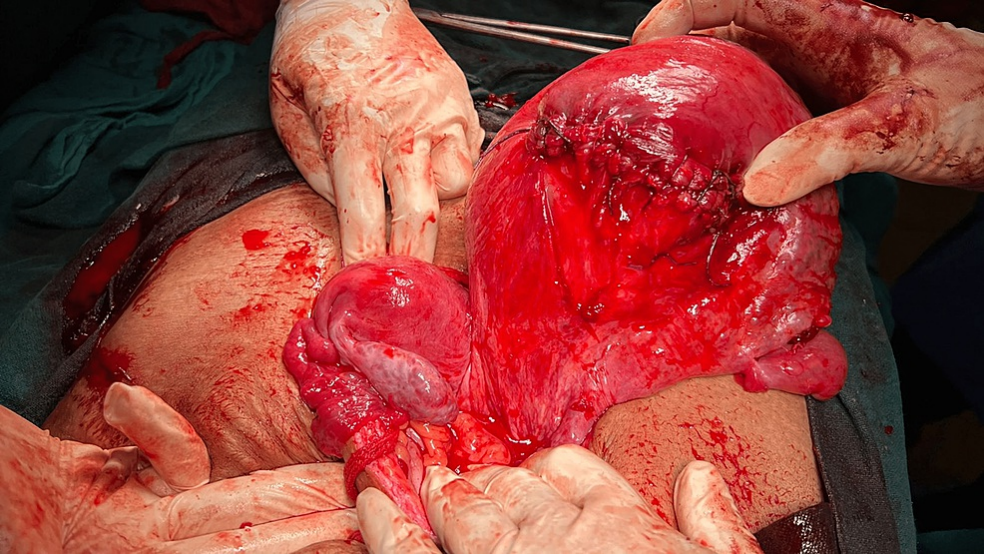
Posterior view of uterus didelphys

There were engorged serpentine blood vessels above these vital structures. Space was created with meticulous planning and the transverse incision was made on the uterus. The baby was successfully delivered by breech extraction after cutting through the placenta. Liquor was clear and the baby had an Appearance Pulse Grimace (reflex) Activity Respiration (APGAR) score of 9 with a birth weight of 3.17 kilograms. After the separation of the placenta, it was observed that due to uterine torsion, the incision was made on the posterior surface of the uterus. Manual untwisting of the uterus was done in the direction opposite to that of the torsion. The uterus had torsion by more than 180 degrees on its own longitudinal axis, at the level of the junction of the cervix, the corpus and the right adnexa was covering the operative site. The posterior surface of the uterus was placed anteriorly due to torsion (Figures 2-3). 

Another intact uterus was found on the left side of the pelvic cavity (about 12 weeks in size) with a tube and ovary attached to it. The final diagnosis was uterus didelphys with torsion of the gravid uterus. The uterine incision was repaired in two layers and anti-adhesive barrier gel was placed posteriorly. After ensuring hemostasis, the abdomen was closed. The post-operative period was uneventful. Both mother and baby were discharged on the third postoperative day.

## Discussion

Uterine torsion is defined as the twisting of the uterus by more than 45 degrees around its long axis at the junction between the cervix and the corpus [2]. Torsion of the uterus is very rare in obstetrics. Malpresentation (particularly transverse lie), myomas, uterine anomalies, pelvic adhesions, ovarian cysts, abnormal pelvis, and placenta may predispose to this condition [3]. Clinical diagnosis is difficult as symptoms may be absent. Diagnosis can only be made intraoperatively. Torsion may cause adverse maternal and neonatal consequences.

Very few cases of torsion in uterus didelphys have been published as of date. Only two cases of torsion of uterus didelphys (one at term pregnancy and the other in labour) were published in 1954 [4] and 1982 [5], respectively. A case report was published after uterine torsion in the puerperium following caesarean section in the uterus didelphys [6].

In our case, malpresentation (transverse lie) and Mullerian anomaly (uterus didelphys) predisposed to torsion of the uterus. The diagnosis of uterus didelphys was missed during the previous Caesarean section which was operated on for fetal distress in emergency hours. During the current pregnancy, the other uterus was thought to be a myoma in ultrasound and was a surprise during surgery. Moreover, the torsion of more than 180 degrees placed the anterior surface of the uterus posteriorly and vice versa. The baby was delivered through a transverse incision on the posterior surface of the uterus.

From the literature review, it has been observed that a pre-operative diagnosis of uterine torsion is not possible. It is also difficult to correct uterine torsion before the delivery of the fetus. Maternal and perinatal morbidity and mortality depend on the degree of rotation of the uterus and the period of gestation.

In this case, prompt intraoperative planning and decision by the surgeon enabled a favourable outcome for both the mother and the baby. 

## Conclusions

In cases of uterine torsion, a prompt and timely decision by the surgeon is crucial. The uterine incision should be planned to avoid injury to vital structures. The diagnosis, though rare, should be kept in mind in all cases of abdominal pain during pregnancy, especially in those with malpresentation. A pelvic mass in pregnancy can rarely be a uterus too in case of Mullerian anomaly.
